# Acute effect of low-dose thiacloprid exposure synergised by tebuconazole in a parasitoid wasp

**DOI:** 10.1371/journal.pone.0212456

**Published:** 2019-02-22

**Authors:** Jonathan Willow, Ana Silva, Eve Veromann, Guy Smagghe

**Affiliations:** 1 Laboratory of Agrozoology, Department of Plants and Crops, Faculty of Bioscience Engineering, Ghent University, Ghent, Belgium; 2 Chair of Plant Health, Institute of Agricultural and Environmental Sciences, Estonian University of Life Sciences, Tartu, Estonia; 3 Cardiff University Brain Research Imaging Centre (CUBRIC), School of Psychology, Cardiff University, Cardiff, United Kingdom; University of California San Diego, UNITED STATES

## Abstract

Agricultural practices often involve tank-mixing and co-application of insecticides with fungicides to control crop pests. However, natural methods relying on biological control agents such as hymenopteran parasitoids have been shown to be highly effective in suppressing crop pest populations. The current body of insecticide risk assessment data accounting for fungicide co-application is very small, the present study being the first to examine this in a parasitoid wasp. Through low-dose exposure to dry residues of the neonicotinoid insecticide thiacloprid, we examined its mortal and knockdown effect on *Aphelinus abdominalis* when co-applied with increasing doses of the fungicide tebuconazole. Both of these acute effects of thiacloprid were synergised (toxicity increased to a greater-than-additive effect) by tebuconazole, resulting in significant mortality from low-dose co-applications of tebuconazole, and significant knockdown even without co-applied tebuconazole, the effect increasing as tebuconazole concentration increased. We show the highly toxic effect that a low dose of thiacloprid imposes on *A*. *abdominalis* populations, and a synergistic toxicity when co-applied with low doses of tebuconazole. Our work suggests a need for updating pesticide risk assessment methods, accounting for pesticide mixtures, in order to make these risk assessments more field relevant.

## Introduction

Insects contribute to several ecosystem services that are indispensable to agriculture [1], one of which is biological control of crop pests. Parasitoid wasps in particular can be very effective at suppressing insect pest populations in agroecosystems [2–10]. However, in conventional agriculture, farmers apply pesticides to manage crop pests, often routinely without regard for pest incidence and abundance, even though research has indicated lethal and sublethal effects of both botanical and synthetic pesticides on numerous parasitoid wasp species of ecological and economic importance [11–24]. Among insecticide classes, chloronicotinyls (neonicotinoids, IRAC class 4A of nicotinic acetylcholine receptor (nAChR) competitive modulators) are especially hazardous for insect populations due to their systemic action in plants, resulting in not only surface contamination from spray residues, but potential contamination of all plant tissues and floral/extrafloral rewards (e.g. nectar, pollen, guttation). Recently in April 2018, after considerable evidence had been gathered regarding adverse effects of these systemic insecticides on beneficial insects [25], all member states of the European Union agreed to ban outdoor use of three neonicotinoid insecticides, namely imidacloprid, clothianidin and thiamethoxam. However, there are 13 neonicotinoid active ingredients patented for use as insecticides [26].

In practice, insecticides are often tank-mixed with fungicides for simultaneous application to agricultural fields [27,28]. The ability of a fungicide to synergise the toxicity of an insecticide has been clearly demonstrated in the honeybee *Apis mellifera* (L.) [29–32], the mason bee *Osmia bicornis* (L.) [33,34] and the bumblebee *Bombus terrestris* (L.) [35]. This means the effect of pyrethroid and neonicotinoid insecticides combined with ergosterol biosynthesis inhibitor fungicides is greater than the sum of each one’s effect when applied individually. The suggested mechanism behind this is that exposure these fungicides inhibits production of cytochrome P450-dependent monooxygenases, enzymes necessary for oxidative metabolism of a variety of xenobiotics including insecticides [36]. The available data demonstrating this phenomenon in non-target insects are currently limited to the above-mentioned three species in the bee superfamily Apoidea. Parasitoid wasps represent another relevant group of hymenopteran insects for examining this phenomenon, their populations being essential for self-sustaining pest control processes and integrated pest management (IPM). In addition to their role as biocontrol agents, their size and behavioural differences compared to the above-mentioned bee species suggests the need for insecticide risk assessment data accounting for fungicide co-application in a parasitoid model.

The neonicotinoid insecticide thiacloprid and the fungicide tebuconazole (FRAC code 3, demethylation inhibitors, class 1 of sterol biosynthesis inhibitors) are both applied, sometimes as tank-mixture [28], for crop protection in a variety of agroecosystems, including but not limited to oilseed rape, wheat, orchards and cotton. The parasitoid wasp family Aphelinidae is an important taxon of parasitoids (primarily of aphids and other Homoptera) distributed across the world, inhabiting almost all habitat types. This diverse family contains approximately 1160 species in 33 genera and 7 subfamilies [37].

Here we exposed the aphelinid wasp *Aphelinus abdominalis* (Dalman), an important biological control agent for suppressing aphid populations, to a low concentration of dry residues of thiacloprid, with and without co-applications of tebuconazole at various concentrations at or below manufacturer’s recommended dose (MRD). After exposure via dry residues on glass surface, we monitored the effect of each treatment on *A*. *abdominalis* mortality (lethal effect) and knockdown (loss of motor control, sublethal effect) over a 24 h period. Based on the findings of above-mentioned analogous research on bees, we expected tebuconazole to synergise these acute effects of thiacloprid in *A*. *abdominalis*, and to observe a threshold-dose of tebuconazole corresponding to this synergy. This work is intended to show the increased lethality and sublethality imposed on *A*. *abdominalis* populations when exposed to multiple pesticides simultaneously, as well as the increased field-relevance of insecticide risk assessment when examining pesticides in combination, since these compounds are tank-mixed and simultaneously applied in agricultural practice.

## Materials and methods

Prior to this study, we explored the use of three different parasitoid wasp species, namely *Aphidius matricariae* (Haliday) (Braconidae), *Diglyphus iseae* (Walker) (Eulophidae) and *Aphelinus abdominalis*. These preliminary tests showed a similar mortality effect of thiacloprid on each species examined (data not shown). We decided to focus on *A*. *abdominalis* due to the ease of handling this species without the need to slow down their activity using cold temperatures.

The two active ingredients examined, and their respective formulations, were thiacloprid [Calypso, active ingredient 480 g/l, suspension concentrate (Bayer CropScience)] and tebuconazole [Tebusip, active ingredient 250 g/l, emulsifiable concentrate (OXON Italia)]. Aphid mummies containing diapausing *A*. *abdominalis* adults, were ordered from Biobest (Westerlo, Belgium), and subsequently maintained in an incubator (22 °C, 60% relative humidity, 16:8 h light:dark; model MLR-352H-PE Climate Chamber, Panasonic, Kadoma, Japan). Insects were used in experiments shortly (1–2 days) after emergence from diapause.

In search of a suitable dose of thiacloprid for examining synergy dynamics, 100 individuals (5 cages of 20 insects) of *A*. *abdominalis* were exposed to dried residues of thiacloprid at MRD (120 g/ha), as well as a control treatment (de-ionized water). The effectiveness of thiacloprid at MRD on knockdown (shown in Results) suggested that this dose was unsuitable for examining synergy dynamics, and thus we subsequently reduced our experimental dose of thiacloprid to one tenth MRD (12 g/ha).

Treatments for the experiment are shown in Table 1. They include an untreated control (de-ionized water), tebuconazole at MRD (125 g/ha), thiacloprid at one tenth MRD, and five treatments containing both thiacloprid and tebuconazole. In combinatory treatments, we applied thiacloprid always at one tenth MRD, while tebuconazole was co-applied at one one-hundredth MRD (1.25 g/ha), one twentieth MRD (6.25 g/ha), one tenth MRD (12.5 g/ha), one half MRD (62.5 g/ha) and MRD (125 g/ha). We used 240 insects (12 cages of 20 insects) per treatment.

**Table 1:** List of treatments in pesticide mixture experiment. H_2_O = control, TH = thiacloprid, TEB = tebuconazole, [0.01] = one one-hundredth manufacturer’s recommended dose (MRD), [0.05] = one twentieth MRD, [0.1] = one tenth MRD, [0.5] = one half MRD, [1] = MRD.

Treatment	Dose of TH (g/ha)	Dose of TEB (g/ha)
H_2_O	0	0
TEB [1]	0	125
TH [0.1]	12	0
TH [0.1] + TEB [0.01]	12	1.25
TH [0.1] + TEB [0.05]	12	6.25
TH [0.1] + TEB [0.1]	12	12.5
TH [0.1] + TEB [0.5]	12	62.5
TH [0.1] + TEB [1]	12	125

For each treatment, circular glass discs (9 cm diameter) were individually loaded into a Cornelis spray tower [38], and sprayed with 1 ml of treatment solution (formulation and de-ionized water) using 1 bar of air pressure. Prior to experiments, treatment solutions were diluted and mixed based on the surface area of the glass plates and the use of 1 ml of solution per spray per disc, in order to apply solutions representing a series of active ingredient concentrations in g/ha based on MRD. To ensure the use of fresh treatments for each experimental repeat, new treatment solutions were prepared weekly, kept in sterile 50 ml polypropylene centrifuge tubes (Nerbe Plus, Germany) and stored in a refrigerator. The treated glass discs were left to air-dry for approximately 2 h, ensuring that only dried treatment residues remained on the plates.

Cages were constructed from two treated glass discs and a plastic ring frame (height of 14 mm) with small holes covered in fine mesh to provide ventilation and prevent internal condensation. For each cage, the plastic ring frame was placed over the perimeter of the bottom disc, which was then fastened to the ring frame, and 20 randomly chosen individuals were introduced to the base of the cage using a fine paintbrush. Then the top disc was placed over the ring frame to complete the cage, and all materials were fastened together. The treated side of each glass disc faced interiorly in each constructed cage. The sex of each wasp was not taken into account, in order to prevent any damage to the insects from too much handling. Finally, as a food source, a strip of filter paper soaked in 50% sucrose solution (blotted with a paper towel to reduce wetness, further preventing internal condensation) was added to the cage through a larger hole in the plastic frame, which was then sealed with a rubber stopper. Cages with wasps were then placed in the incubator.

Using a 14x magnification hand lens, both mortality and knockdown were recorded at 2, 4, 6, 8 and 24 h post-exposure. Mortality was assumed when an individual showed no movement (14x magnification) during 15 s of observation, even after gently prodding and stroking the insect with a fine paintbrush. Knockdown was determined when erratic muscular activity (stumbling, convulsing) or a lack of muscular activity (partial or entire paralysis) inhibited an individual from moving about in a stable manner or at all, taking into account all situations where an individual was incapable of performing biological control services (including apparent mortality, therefore representing total acute effect). Knockdown is an important endpoint to examine, as a very small insect like *A*. *abdominalis* may likely die shortly after knockdown in natural situations due to various factors (e.g. dehydration, predation, inability to forage).

Statistical analyses were performed with R software (version 1.0.136) [39]. We used a one-way ANOVA followed by *post-hoc* pairwise comparisons using two-tailed unpaired t-tests and corrected for multiple comparisons using the Bonferroni correction method. Since the residuals of the linear model were normal, we used non-transformed data. Synergistic toxicity of pesticide mixture treatments was determined by subtracting single-compound effects, of both thiacloprid (Effect_TH_) and tebuconazole (Effect_TEB_), from the effect of a given combinatory treatment (Effect_TH+TEB_). An Effect_TH+TEB_ greater than the combined sum of Effect_TH_ and Effect_TEB_ indicates synergistic toxicity.

## Results

**Fig 1:**
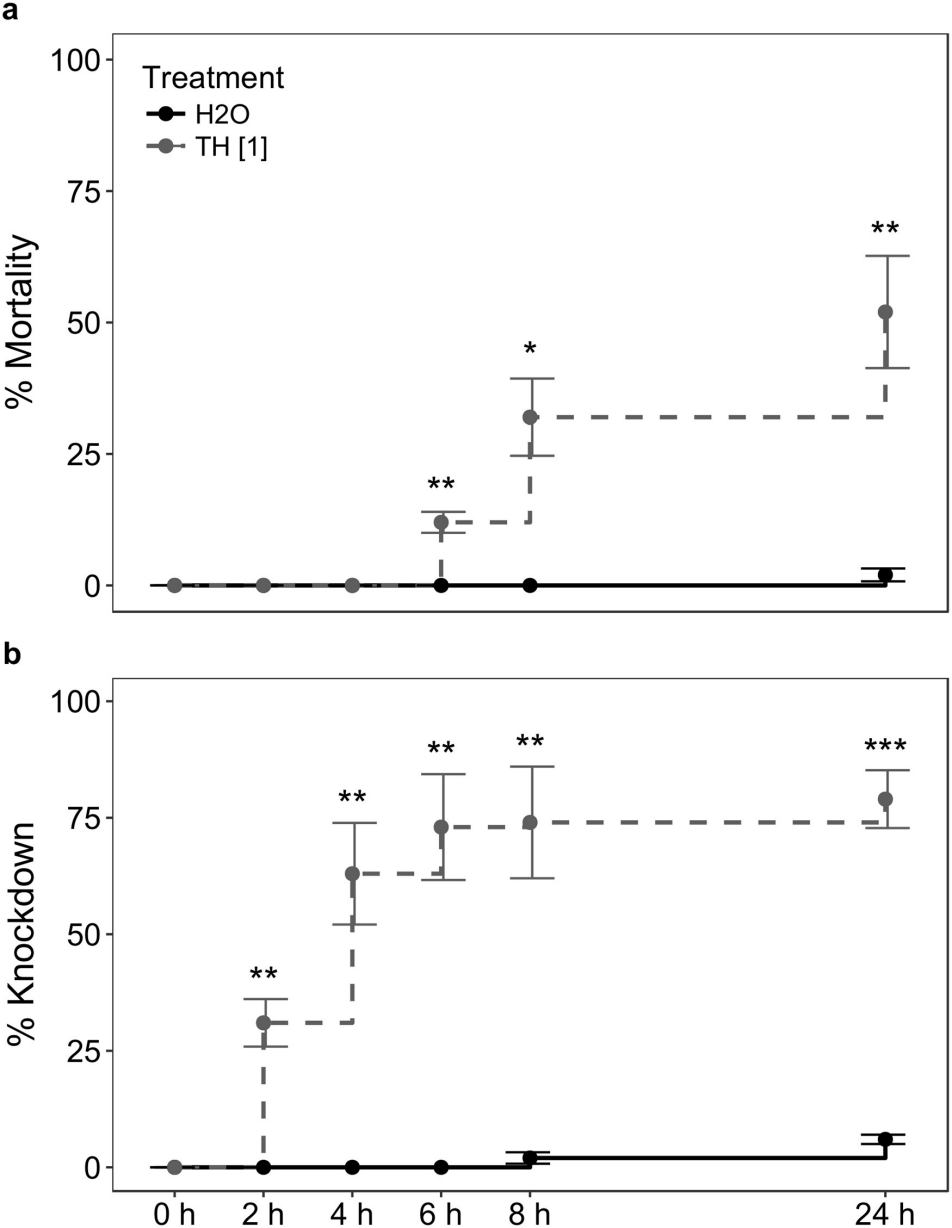
Effect of thiacloprid at manufacturer’s recommended dose (MRD, 120 g/ha) on a) mortality, and b) knockdown of the parasitoid wasp *Aphelinus abdominalis* at different hours after treatment (error bars: ±SEM). **N = 100 (5 cages of 20 insects)**. H_2_O = control, TH = thiacloprid. Welch two-tailed unpaired t-test: * = p<0.05, ** = p<0.01, *** = p<0.001.

When examining the effect of thiacloprid at MRD on *A*. *abdominalis*, after 24 h we observed 52% mortality (p = 0.009, Welch two-tailed unpaired t-test) and 79% knockdown (p = 0.0002, Welch two-tailed unpaired t-test) from thiacloprid at MRD (Table 2, Fig 1, see S1 and S2 Tables for raw data), showing that this concentration of thiacloprid was too effective on knockdown to reliably use MRD of thiacloprid for our experiment.

**Table 2:** Effect of thiacloprid at manufacturer’s recommended dose (MRD, 120 g(ha)^-1^) on mortality and knockdown of the parasitoid wasp *Aphelinus abdominalis* at different hours after treatment. **N = 100 (5 cages of 20 insects) per treatment**. H_2_O = control, TH = thiacloprid, [1] = manufacturer’s recommended dose (MRD).

Mortality			
t-test (H2O vs TH [1]	t	df	p-value
2 h			
4 h			
6 h	6	4	0.004
8 h	4.35	4	0.01
24 h	4.65	4.11	0.009
Knockdown			
t-test (H2O vs TH [1]	t	df	p-value
2 h	6.08	4	0.004
4 h	5.78	4	0.004
6 h	6.43	4	0.003
8 h	5.98	4.08	0.004
24 h	11.62	4.21	0.0002

**Fig 2:**
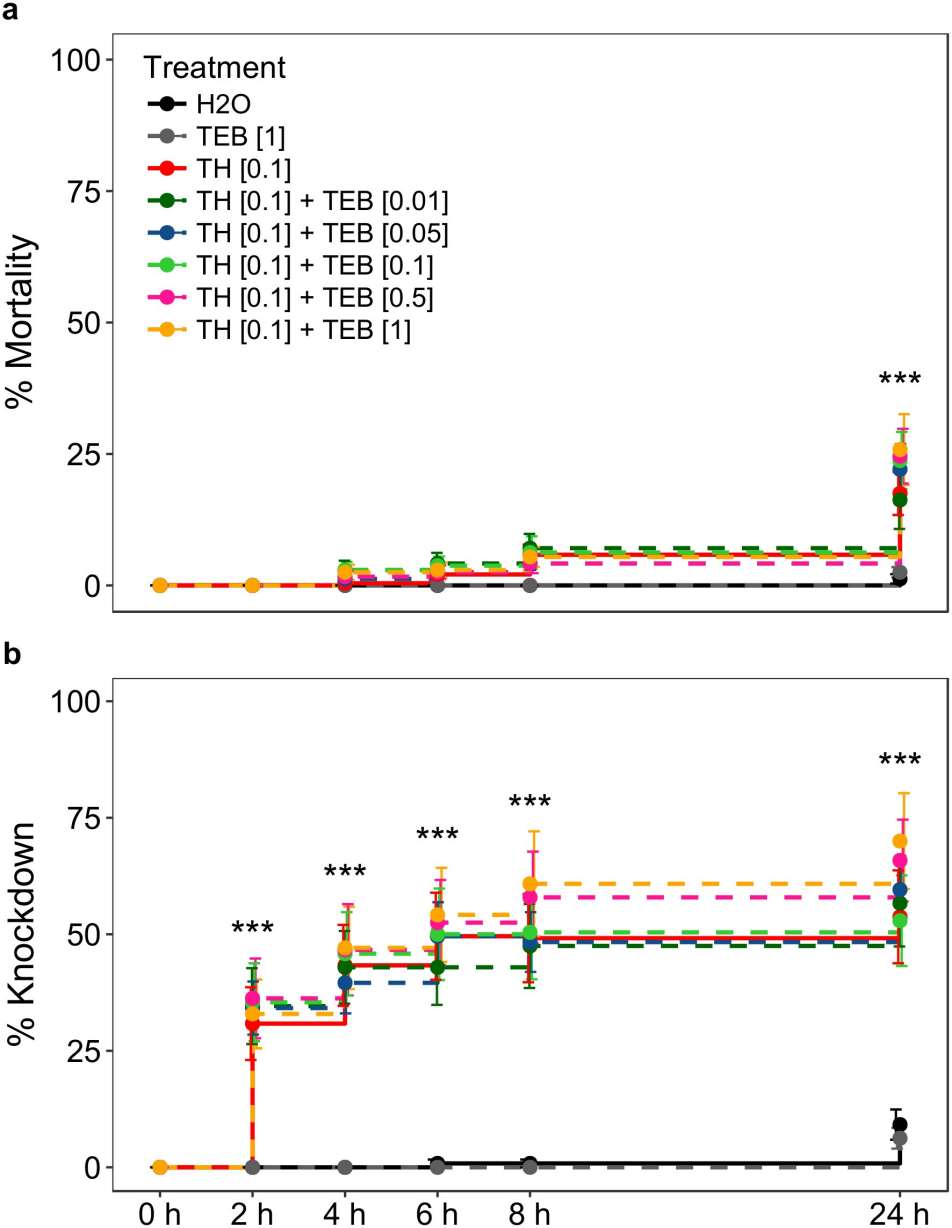
Effect of each treatment on a) mortality and b) knockdown of the parasitoid wasp *Aphelinus abdominalis* at different hours after treatment (error bars: ±SEM). **N = 240 (12 cages of 20 insects) per treatment**. H_2_O = control, TH = thiacloprid, TEB = tebuconazole, [0.01] = one one-hundredth manufacturer’s recommended dose (MRD), [0.05] = one twentieth MRD, [0.1] = one tenth MRD, [0.5] = one half MRD, [1] = MRD. One-way ANOVA: * = p<0.05, ** = p<0.01, *** = p<0.001.

When exposing *A*. *abdominalis* to all 8 treatments in our examination of thiacloprid at one tenth MRD combined with increasing doses of tebuconazole (N = 240 per treatment), we observed tebuconazole’s synergising effect on thiacloprid (effect of combinatory treatments were greater than the summed effect of both thiacloprid and tebuconazole by themselves), with regard to mortality at 24 h for combinatory treatments containing tebuconazole at one twentieth MRD, one tenth MRD, one half MRD and MRD (Fig 2a). F statistics, assessed using one-way ANOVA, showed significant lethal effects, at 24 h, of using different treatments (Table 3, Fig 2a, see S3 Table for raw data).

**Table 3:** Results from analysis of variance (ANOVA) for the effect of the different treatments on mortality and knockdown of the parasitoid wasp *Aphelinus abdominalis* in each cage (12 cages, each with 20 insects) at different hours after treatment. dfn = degrees of freedom numerator, dfd = degrees of freedom denominator.

Time	dfn, dfd	F-statistics	Pr (>F)
Mortality			
2 h	(7,88)	1	0.44
4 h	(7,88)	1.28	0.27
6 h	(7,88)	1.53	0.17
8 h	(7,88)	2.05	0.06
24 h	(7,88)	4.37	<0.001
Knockdown			
2 h	(7,88)	5.62	<0.0001
4 h	(7.88)	7.86	<0.0001
6 h	(7.88)	8.73	<0.0001
8 h	(7.88)	8.95	<0.0001
24 h	(7,88)	9.28	<0.0001

*Post-hoc* pairwise comparisons revealed that thiacloprid at one tenth MRD by itself did not significantly affect mortality at 24 h (p = 0.44) (Table 4) compared to the control (H_2_O). However, a significant effect on mortality resulted from combining thiacloprid at one tenth MRD with tebuconazole at one tenth MRD (p = 0.03), one half MRD (p = 0.02) and MRD (p<0.01), as well as a marginally significant effect when combined with tebuconazole at one twentieth MRD (p = 0.06). We observed a trend of increased mortality as the concentration of tebuconazole in the binary mixture increased.

**Table 4:** Results from *post-hoc* pairwise comparisons (two-tailed unpaired t-test) between the control and each other treatment, for both mortality and knockdown. H_2_O = control, TH = thiacloprid, TEB = tebuconazole, [0.01] = one one-hundredth manufacturer’s recommended dose (MRD), [0.05] = one twentieth MRD, [0.1] = one tenth MRD, [0.5] = one half MRD, [1] = MRD. All p-values shown have been adjusted for multiple comparisons using Bonferroni correction method.

Treatment vs H_2_O	p-value (Bonferroni)	
Mortality		24 h
TEB [1]		1
TH [0.1]		0.44
TH [0.1] + TEB [0.01]		0.71
TH [0.1] + TEB [0.05]		0.06
TH [0.1] + TEB [0.1]		0.03
TH [0.1] + TEB [0.5]		0.02
TH [0.1] + TEB [1]		0.009
Knockdown	2h	24 h
TEB [1]	1	1
TH [0.1]	0.04	0.006
TH [0.1] + TEB [0.01]	0.01	0.002
TH [0.1] + TEB [0.05]	0.01	0.0009
TH [0.1] + TEB [0.1]	0.009	0.007
TH [0.1] + TEB [0.5]	0.007	0.0001
TH [0.1] + TEB [1]	0.02	< 0.0001

Regarding knockdown, we observed that tebuconazole synergised the effect of thiacloprid at 2, 4, 6, 8 and 24 h in combinatory treatments containing tebuconazole at one half MRD and MRD (Fig 2b, see S4 Table for raw data). F statistics, assessed using one-way ANOVA, showed significant knockdown effects, at 2, 4, 6 8 and 24 h, of using different treatments (Table 3, Fig 2b). *Post-hoc* pairwise comparisons revealed a significant difference in knockdown at 2 h for all treatments containing thiacloprid (Table 4). At 24 h, we observed increased knockdown in all treatments. Knockdown was significant for the treatment containing only thiacloprid at one tenth MRD (p<0.01), as well as the combinatory treatments containing tebuconazole at one one-hundredth MRD (p<0.005), one twentieth MRD (p<0.001), one tenth MRD (p<0.01), one half MRD (p = 0.0001) and MRD (p<0.0001) (Table 4). As with mortality, we observed a trend of increased knockdown as the concentration of tebuconazole in combinatory treatments increased.

## Discussion

Using the parasitoid *wasp A*. *abdominalis* exposed to field-realistic doses of dry pesticide residues, our study provides evidence of an insecticide’s acute lethal and sublethal effect synergised by co-application of a fungicide, the two compounds being commonly tank-mixed for simultaneous use in a variety of agroecosystems [28]. To our knowledge, these are the first data showing this type of agrochemical synergy occurring in a biological control insect.

We observed a synergising of thiacloprid’s effect, by co-exposure to tebuconazole, on mortality for all combinatory treatments containing tebuconazole at one twentieth MRD or higher. We did not observe significant mortality at 24 h when exposing *A*. *abdominalis* to dried residues of thiacloprid at one tenth MRD by itself. However, when thiacloprid at one tenth MRD was co-applied with tebuconazole at one tenth MRD or higher, significant mortality was observed. Our observations on knockdown further revealed the potential harm that tank-mixing thiacloprid and tebuconazole can impose on *A*. *abdominalis* populations. We observed a trend towards increased knockdown as the concentration of tebuconazole increased in combinatory treatments. When thiacloprid at one tenth MRD was co-applied with tebuconazole at one half MRD or higher, a greater-than-additive knockdown effect of combining these two pesticides was consistently observed over 24 h. Thiacloprid at one tenth MRD by itself resulted in significant knockdown of *A*. *abdominalis*, and this effect increased as the co-applied dose of tebuconazole increased. Our results suggest that even when field-applied thiacloprid residues are degraded to a potency one order of magnitude less than application rate, or even if thiacloprid is applied at lower doses such as one tenth MRD, this may cause significant reductions in populations of *A*. *abdominalis* or similar species, especially when thiacloprid is tank-mixed with tebuconazole or perhaps other fungicides. Fungicide application to crops containing aged residues of thiacloprid may represent an equal or greater issue, as 14 day old thiacloprid residues on leaves were shown to cause significant mortality in the braconid wasp *Aphidius rhopalosiphi* (De Stefani Perez) [40].

As endpoints, the present study examined mortality and knockdown, both being appropriate and relevant. Indeed, knockdown may be a more accurate estimation, compared to mortality, of the damage these agrochemicals can inflict on a population, since insects that lack or simply cannot control their motor activity are highly unlikely to successfully perform the ecological services for which they are revered. Moreover, very small insects like *A*. *abdominalis* may indeed be likely to die not long after knockdown, simply from environmental exposure (e.g. dehydration, predation) and inability to forage. Adding to the concern is a body of evidence indicating that key processes involved in achieving their ecological function (e.g. mate and host recognition, maximising fecundity, optimising offspring sex ratio) can be significantly hindered by exposure to both botanical and synthetic pesticides [11,12,14–18,20,21,25]. It would be beneficial for future studies to investigate how these processes are affected by exposure to pesticide combinations used in agricultural practice.

Our study comes at an appropriate time, as three neonicotinoid insecticides were recently banned for outdoor use in all European Union member states. Thiacloprid was not one of these, and therefore it will be increasingly important to study the lethal and sublethal effects of the neonicotinoids that remain in use. With the new ban on imidacloprid, clothianidin and thiamethoxam, remaining neonicotinoids such as thiacloprid are indeed likely to increase in use.

Results of our study are consistent with the results of Sugiyama and colleagues [13] who exposed three other species of the parasitoid wasp family Aphelinidae to thiacloprid residues. They observed very high mortality rates in *Eretmocerus eremicus* (Rose and Zolnerowich) (98%) and *Encarsia formosa* (Gahan) (86%), and 37% mortality in *Eretmocerus mundus* (Mercet), while in our preliminary test with thiacloprid at MRD we observed 52% mortality and 79% knockdown for *A*. *abdominalis*. Furthermore, a potentially detrimental knockdown effect (an effect which typically precedes death) of thiacloprid, when applied at doses as low as one tenth MRD, is indicated by our results. Sugiyama et al. [13] also examined the lethal effect of five other neonicotinoids (acetamiprid, clothianidin, dinotefuran, imidacloprid and nitenpyram) in the same study, and observed 100% mortality in all three aphelinid species for all five of these insecticides. Our work, combined with that of Sugiyama et al. [13], suggests that exposure to thiacloprid residues may be detrimental to numerous parasitoid wasp species of the family Aphelinidae. To confirm this, however, would require exhaustive research within the context of this species-rich family.

The present study examined the effect of contact with dried pesticide residues. Under field conditions, multiple routes of exposure to these systemic compounds are likely to occur simultaneously (e.g. direct exposure to spray droplets; contact with dried surface residues; larval feeding on contaminated prey; and adult feeding on contaminated nectar, pollen, honeydew and guttation). Accounting for these various routes of pesticide exposure is encouraged for further studies. Furthermore, pesticide exposure is poorly understood for many non-target invertebrate taxa, and it is needed that experiments simulate realistic exposure. Mesocosm experiments simulating natural community exposure to relevant pesticide combinations [41] are also encouraged.

Our results suggest that the degree of tebuconazole’s synergising effect on thiacloprid depends on the dose of co-applied tebuconazole. Enzyme assays confirming cytochrome P450-dependent monooxygenase inhibition, and its response to increasing or decreasing co-applied doses of tebuconazole, could increase our understanding of how these compounds act together, a prerequisite to informed use of these agrochemicals.

We examined the effect on mortality and knockdown, accounting for any instance of clear incapability to perform biological control services. Examining other sublethal effects (e.g. on reproduction, microbiome, feeding, longevity, etc.) could add much to our understanding of how these field-applied pesticide mixtures may affect population sustenance, and should be incorporated into further studies. In order to increase field realism, further studies should also incorporate the use of treated live plants as the contaminated experimental surface, as unlike glass these living tissues are absorptive to the liquid treatments examined here. In addition, although our treatments were prepared weekly and kept refrigerated in the dark, pesticide formulations are best used immediately after preparation/dilution.

The synergising of thiacloprid’s acute effect, via co-application of tebuconazole, as shown in our study, suggests the necessity for updating the standards by which we perform insecticide risk assessments on non-target organisms, by including other pesticides with which these insecticides are commonly tank-mixed and co-applied, promoting increased field relevance in risk assessments.

## Supporting information

S1 Table Raw data showing numbers of dead *Aphelinus abdominalis* per cage (thiacloprid vs H_2_O). (DOCX)

S2 Table Raw data showing numbers of knocked down *Aphelinus abdominalis* per cage (thiacloprid vs H_2_O). (DOCX)

S3 Table Raw data showing numbers of dead *Aphelinus abdominalis* per cage for all treatments. (DOCX)

S4 Table Raw data showing numbers of knocked down *Aphelinus abdominalis* per cage for all treatments. (DOCX)
