# Supplementary material for: Acute effect of low-dose thiacloprid exposure synergised by tebuconazole in a parasitoid wasp
Source: PLoS One. 2019 Feb 22;14(2):e0212456. doi: 10.1371/journal.pone.0212456 (PMC6386243; doi:10.1371/journal.pone.0212456)
Supplement: S1 Table — (DOCX) [file pone.0212456.s001.docx]

**S1 Table. Raw data showing numbers of dead Aphelinus abdominalis per cage. N=100 (5 cages of 20 insects).** H_2_O=control, TH=thiacloprid, [1]=manufacturer’s recommended dose (MRD).

| Treatment | Cage no. | 2 h | 4 h | 6 h | 8 h | 24 h |
| --- | --- | --- | --- | --- | --- | --- |
| H_2_O | 1 | 0 | 0 | 0 | 0 | 1 |
|  | 2 | 0 | 0 | 0 | 0 | 1 |
|  | 3 | 0 | 0 | 0 | 0 | 0 |
|  | 4 | 0 | 0 | 0 | 0 | 0 |
|  | 5 | 0 | 0 | 0 | 0 | 0 |
| TH [1] | 1 | 0 | 0 | 3 | 3 | 2 |
|  | 2 | 0 | 0 | 3 | 7 | 12 |
|  | 3 | 0 | 0 | 3 | 10 | 14 |
|  | 4 | 0 | 0 | 1 | 9 | 12 |
|  | 5 | 0 | 0 | 2 | 3 | 12 |
