# Supplementary material for: Acute effect of low-dose thiacloprid exposure synergised by tebuconazole in a parasitoid wasp
Source: PLoS One. 2019 Feb 22;14(2):e0212456. doi: 10.1371/journal.pone.0212456 (PMC6386243; doi:10.1371/journal.pone.0212456)
Supplement: S2 Table — (DOCX) [file pone.0212456.s002.docx]

**S2 Table. Raw data showing numbers of knocked down Aphelinus abdominalis per cage. N=100 (5 cages of 20 insects).** H_2_O=control, TH=thiacloprid, [1]=manufacturer’s recommended dose (MRD).

| Treatment | Cage no. | 2 h | 4 h | 6 h | 8 h | 24 h |
| --- | --- | --- | --- | --- | --- | --- |
| H2O | 1 | 0 | 0 | 0 | 0 | 2 |
|  | 2 | 0 | 0 | 0 | 0 | 1 |
|  | 3 | 0 | 0 | 0 | 1 | 1 |
|  | 4 | 0 | 0 | 0 | 1 | 1 |
|  | 5 | 0 | 0 | 0 | 0 | 1 |
| TH [1] | 1 | 4 | 4 | 6 | 6 | 11 |
|  | 2 | 10 | 14 | 14 | 14 | 16 |
|  | 3 | 5 | 16 | 18 | 19 | 18 |
|  | 4 | 6 | 15 | 18 | 19 | 17 |
|  | 5 | 6 | 14 | 17 | 16 | 17 |
