# Supplementary material for: Acute effect of low-dose thiacloprid exposure synergised by tebuconazole in a parasitoid wasp
Source: PLoS One. 2019 Feb 22;14(2):e0212456. doi: 10.1371/journal.pone.0212456 (PMC6386243; doi:10.1371/journal.pone.0212456)
Supplement: S3 Table — (DOCX) [file pone.0212456.s003.docx]

**S3 Table. Raw data showing numbers of dead Aphelinus abdominalis per cage for all treatments. N=240 (12 cages of 20 insects) per treatment.** H_2_O=control, TH=thiacloprid, TEB=tebuconazole, [0.01]=one one-hundredth manufacturer’s recommended dose (MRD), [0.05]=one twentieth MRD, [0.1]=one tenth MRD, [0.5]=one half MRD, [1]=MRD.

| Treatment | Cage no. | 2 h | 4 h | 6 h | 8 h | 24 h |
| --- | --- | --- | --- | --- | --- | --- |
| H_2_O | 1 | 0 | 0 | 0 | 0 | 0 |
|  | 2 | 0 | 0 | 0 | 0 | 0 |
|  | 3 | 0 | 0 | 0 | 0 | 0 |
|  | 4 | 0 | 0 | 0 | 0 | 0 |
|  | 5 | 0 | 0 | 0 | 0 | 0 |
|  | 6 | 0 | 0 | 0 | 0 | 1 |
|  | 7 | 0 | 0 | 0 | 0 | 0 |
|  | 8 | 0 | 0 | 0 | 0 | 0 |
|  | 9 | 0 | 0 | 0 | 0 | 0 |
|  | 10 | 0 | 0 | 0 | 0 | 0 |
|  | 11 | 0 | 0 | 0 | 0 | 2 |
|  | 12 | 0 | 0 | 0 | 0 | 0 |
| TEB [1] | 1 | 0 | 0 | 0 | 0 | 0 |
|  | 2 | 0 | 0 | 0 | 0 | 0 |
|  | 3 | 0 | 0 | 0 | 0 | 0 |
|  | 4 | 0 | 0 | 0 | 0 | 0 |
|  | 5 | 0 | 0 | 0 | 0 | 0 |
|  | 6 | 0 | 0 | 0 | 0 | 1 |
|  | 7 | 0 | 0 | 0 | 0 | 1 |
|  | 8 | 0 | 0 | 0 | 0 | 2 |
|  | 9 | 0 | 0 | 0 | 0 | 0 |
|  | 10 | 0 | 0 | 0 | 0 | 0 |
|  | 11 | 0 | 0 | 0 | 0 | 1 |
|  | 12 | 0 | 0 | 0 | 0 | 1 |
| TH [0.1] | 1 | 0 | 0 | 0 | 1 | 2 |
|  | 2 | 0 | 0 | 1 | 4 | 6 |
|  | 3 | 0 | 0 | 1 | 2 | 5 |
|  | 4 | 0 | 0 | 1 | 1 | 4 |
|  | 5 | 0 | 0 | 1 | 2 | 4 |
|  | 6 | 0 | 0 | 0 | 0 | 5 |
|  | 7 | 0 | 0 | 0 | 3 | 8 |
|  | 8 | 0 | 1 | 1 | 1 | 7 |
|  | 9 | 0 | 0 | 0 | 0 | 0 |
|  | 10 | 0 | 0 | 0 | 0 | 1 |
|  | 11 | 0 | 0 | 0 | 0 | 0 |
|  | 12 | 0 | 0 | 0 | 0 | 0 |
| TH [0.1] + TEB [0.01] | 1 | 0 | 0 | 0 | 0 | 0 |
|  | 2 | 0 | 1 | 2 | 4 | 5 |
|  | 3 | 0 | 0 | 0 | 1 | 1 |
|  | 4 | 0 | 0 | 0 | 0 | 2 |
|  | 5 | 0 | 0 | 0 | 1 | 5 |
|  | 6 | 0 | 0 | 1 | 2 | 6 |
|  | 7 | 0 | 2 | 4 | 5 | 13 |
|  | 8 | 0 | 4 | 3 | 4 | 5 |
|  | 9 | 0 | 0 | 0 | 0 | 0 |
|  | 10 | 0 | 0 | 0 | 0 | 0 |
|  | 11 | 0 | 0 | 0 | 0 | 1 |
|  | 12 | 0 | 0 | 0 | 0 | 1 |
| TH [0.1] + TEB [0.05] | 1 | 0 | 0 | 1 | 1 | 3 |
|  | 2 | 0 | 0 | 1 | 1 | 1 |
|  | 3 | 0 | 1 | 1 | 2 | 4 |
|  | 4 | 0 | 0 | 0 | 0 | 1 |
|  | 5 | 0 | 0 | 0 | 1 | 7 |
|  | 6 | 0 | 0 | 0 | 2 | 7 |
|  | 7 | 0 | 1 | 2 | 2 | 11 |
|  | 8 | 0 | 1 | 1 | 1 | 8 |
|  | 9 | 0 | 0 | 0 | 0 | 2 |
|  | 10 | 0 | 0 | 0 | 0 | 6 |
|  | 11 | 0 | 0 | 0 | 0 | 1 |
|  | 12 | 0 | 0 | 0 | 0 | 2 |
| TH [0.1] + TEB [0.1] | 1 | 0 | 0 | 0 | 0 | 1 |
|  | 2 | 0 | 0 | 0 | 0 | 7 |
|  | 3 | 0 | 2 | 3 | 6 | 10 |
|  | 4 | 0 | 0 | 0 | 0 | 7 |
|  | 5 | 0 | 0 | 0 | 0 | 6 |
|  | 6 | 0 | 0 | 1 | 2 | 10 |
|  | 7 | 0 | 2 | 2 | 2 | 5 |
|  | 8 | 0 | 3 | 3 | 5 | 8 |
|  | 9 | 0 | 0 | 0 | 0 | 1 |
|  | 10 | 0 | 0 | 0 | 0 | 1 |
|  | 11 | 0 | 0 | 0 | 0 | 1 |
|  | 12 | 0 | 0 | 0 | 0 | 0 |
| TH [0.1] + TEB [0.5] | 1 | 0 | 0 | 0 | 0 | 1 |
|  | 2 | 0 | 0 | 2 | 3 | 3 |
|  | 3 | 0 | 0 | 0 | 2 | 9 |
|  | 4 | 0 | 0 | 0 | 0 | 7 |
|  | 5 | 0 | 0 | 0 | 0 | 9 |
|  | 6 | 0 | 0 | 0 | 0 | 6 |
|  | 7 | 0 | 2 | 2 | 3 | 9 |
|  | 8 | 0 | 2 | 2 | 2 | 9 |
|  | 9 | 0 | 0 | 0 | 0 | 0 |
|  | 10 | 0 | 0 | 0 | 0 | 3 |
|  | 11 | 0 | 0 | 0 | 0 | 1 |
|  | 12 | 0 | 0 | 0 | 0 | 2 |
| TH [0.1] + TEB [1] | 1 | 0 | 0 | 0 | 1 | 0 |
|  | 2 | 0 | 1 | 1 | 1 | 8 |
|  | 3 | 0 | 0 | 0 | 2 | 7 |
|  | 4 | 0 | 0 | 0 | 0 | 6 |
|  | 5 | 0 | 0 | 0 | 1 | 7 |
|  | 6 | 0 | 0 | 1 | 1 | 8 |
|  | 7 | 0 | 3 | 3 | 3 | 14 |
|  | 8 | 0 | 2 | 2 | 4 | 10 |
|  | 9 | 0 | 0 | 0 | 0 | 0 |
|  | 10 | 0 | 0 | 0 | 0 | 1 |
|  | 11 | 0 | 0 | 0 | 0 | 0 |
|  | 12 | 0 | 0 | 0 | 0 | 1 |
