# Supplementary material for: Acute effect of low-dose thiacloprid exposure synergised by tebuconazole in a parasitoid wasp
Source: PLoS One. 2019 Feb 22;14(2):e0212456. doi: 10.1371/journal.pone.0212456 (PMC6386243; doi:10.1371/journal.pone.0212456)
Supplement: S4 Table — (DOCX) [file pone.0212456.s004.docx]

**S4 Table. Raw data showing numbers of knocked down Aphelinus abdominalis per cage for all treatments. N=240 (12 cages of 20 insects) per treatment.** H_2_O=control, TH=thiacloprid, TEB=tebuconazole, [0.01]=one one-hundredth manufacturer’s recommended dose (MRD), [0.05]=one twentieth MRD, [0.1]=one tenth MRD, [0.5]=one half MRD, [1]=MRD.

| Treatment | Cage no. | 2 h | 4 h | 6 h | 8 h | 24 h |
| --- | --- | --- | --- | --- | --- | --- |
| H_2_O | 1 | 0 | 0 | 0 | 0 | 0 |
|  | 2 | 0 | 0 | 0 | 0 | 1 |
|  | 3 | 0 | 0 | 0 | 0 | 0 |
|  | 4 | 0 | 0 | 0 | 0 | 0 |
|  | 5 | 0 | 0 | 0 | 0 | 0 |
|  | 6 | 0 | 0 | 2 | 2 | 3 |
|  | 7 | 0 | 0 | 0 | 0 | 6 |
|  | 8 | 0 | 0 | 0 | 0 | 5 |
|  | 9 | 0 | 0 | 0 | 0 | 0 |
|  | 10 | 0 | 0 | 0 | 0 | 0 |
|  | 11 | 0 | 0 | 0 | 0 | 4 |
|  | 12 | 0 | 0 | 0 | 0 | 3 |
| TEB [1] | 1 | 0 | 0 | 0 | 0 | 0 |
|  | 2 | 0 | 0 | 0 | 0 | 1 |
|  | 3 | 0 | 0 | 0 | 0 | 0 |
|  | 4 | 0 | 0 | 0 | 0 | 0 |
|  | 5 | 0 | 0 | 0 | 0 | 0 |
|  | 6 | 0 | 0 | 0 | 0 | 1 |
|  | 7 | 0 | 0 | 0 | 0 | 3 |
|  | 8 | 0 | 0 | 0 | 0 | 4 |
|  | 9 | 0 | 0 | 0 | 0 | 0 |
|  | 10 | 0 | 0 | 0 | 0 | 0 |
|  | 11 | 0 | 0 | 0 | 0 | 3 |
|  | 12 | 0 | 0 | 0 | 0 | 3 |
| TH [0.1] | 1 | 6 | 9 | 9 | 8 | 11 |
|  | 2 | 16 | 19 | 19 | 19 | 18 |
|  | 3 | 7 | 11 | 10 | 11 | 11 |
|  | 4 | 7 | 9 | 11 | 10 | 12 |
|  | 5 | 3 | 10 | 16 | 17 | 16 |
|  | 6 | 2 | 6 | 13 | 16 | 15 |
|  | 7 | 13 | 14 | 16 | 16 | 20 |
|  | 8 | 13 | 17 | 16 | 12 | 17 |
|  | 9 | 6 | 5 | 6 | 6 | 1 |
|  | 10 | 1 | 3 | 2 | 2 | 5 |
|  | 11 | 0 | 0 | 0 | 0 | 1 |
|  | 12 | 0 | 1 | 1 | 1 | 2 |
| TH [0.1] + TEB [0.01] | 1 | 1 | 8 | 7 | 7 | 9 |
|  | 2 | 9 | 15 | 17 | 19 | 16 |
|  | 3 | 10 | 10 | 9 | 9 | 17 |
|  | 4 | 5 | 6 | 8 | 13 | 14 |
|  | 5 | 4 | 8 | 14 | 15 | 17 |
|  | 6 | 9 | 11 | 11 | 13 | 17 |
|  | 7 | 17 | 16 | 14 | 13 | 16 |
|  | 8 | 17 | 17 | 14 | 16 | 17 |
|  | 9 | 3 | 4 | 3 | 4 | 4 |
|  | 10 | 5 | 5 | 5 | 4 | 1 |
|  | 11 | 3 | 3 | 1 | 0 | 5 |
|  | 12 | 0 | 0 | 0 | 1 | 3 |
| TH [0.1] + TEB [0.05] | 1 | 5 | 8 | 11 | 10 | 11 |
|  | 2 | 6 | 8 | 11 | 12 | 12 |
|  | 3 | 10 | 10 | 13 | 11 | 14 |
|  | 4 | 4 | 4 | 6 | 10 | 13 |
|  | 5 | 9 | 8 | 14 | 12 | 14 |
|  | 6 | 8 | 14 | 14 | 11 | 18 |
|  | 7 | 14 | 15 | 17 | 16 | 18 |
|  | 8 | 10 | 10 | 13 | 13 | 15 |
|  | 9 | 8 | 10 | 9 | 9 | 10 |
|  | 10 | 7 | 7 | 9 | 10 | 13 |
|  | 11 | 0 | 0 | 1 | 1 | 3 |
|  | 12 | 1 | 1 | 1 | 1 | 2 |
| TH [0.1] + TEB [0.1] | 1 | 13 | 13 | 10 | 10 | 11 |
|  | 2 | 5 | 9 | 9 | 10 | 11 |
|  | 3 | 16 | 15 | 16 | 16 | 14 |
|  | 4 | 10 | 16 | 17 | 18 | 18 |
|  | 5 | 5 | 12 | 16 | 15 | 17 |
|  | 6 | 9 | 13 | 16 | 17 | 18 |
|  | 7 | 13 | 16 | 13 | 14 | 11 |
|  | 8 | 12 | 11 | 17 | 16 | 18 |
|  | 9 | 1 | 1 | 2 | 2 | 3 |
|  | 10 | 1 | 1 | 3 | 2 | 2 |
|  | 11 | 0 | 1 | 0 | 0 | 1 |
|  | 12 | 0 | 2 | 1 | 1 | 3 |
| TH [0.1] + TEB [0.5] | 1 | 3 | 7 | 10 | 13 | 17 |
|  | 2 | 0 | 1 | 6 | 7 | 9 |
|  | 3 | 11 | 11 | 14 | 17 | 18 |
|  | 4 | 6 | 7 | 8 | 10 | 14 |
|  | 5 | 15 | 19 | 19 | 19 | 18 |
|  | 6 | 13 | 19 | 17 | 19 | 19 |
|  | 7 | 14 | 14 | 16 | 18 | 19 |
|  | 8 | 14 | 18 | 19 | 19 | 20 |
|  | 9 | 7 | 6 | 7 | 6 | 6 |
|  | 10 | 4 | 7 | 7 | 8 | 8 |
|  | 11 | 0 | 2 | 2 | 2 | 5 |
|  | 12 | 0 | 1 | 1 | 1 | 5 |
| TH [0.1] + TEB [1] | 1 | 7 | 12 | 15 | 16 | 19 |
|  | 2 | 9 | 12 | 13 | 16 | 20 |
|  | 3 | 9 | 11 | 13 | 17 | 18 |
|  | 4 | 12 | 15 | 17 | 18 | 20 |
|  | 5 | 3 | 11 | 13 | 17 | 17 |
|  | 6 | 11 | 14 | 17 | 16 | 17 |
|  | 7 | 13 | 16 | 16 | 19 | 19 |
|  | 8 | 12 | 16 | 19 | 20 | 20 |
|  | 9 | 0 | 1 | 1 | 1 | 4 |
|  | 10 | 3 | 3 | 3 | 3 | 5 |
|  | 11 | 0 | 2 | 3 | 2 | 6 |
|  | 12 | 0 | 0 | 0 | 1 | 3 |
